# Current Trends in Automotive Lightweighting Strategies and Materials

**DOI:** 10.3390/ma14216631

**Published:** 2021-11-03

**Authors:** Frank Czerwinski

**Affiliations:** CanmetMATERIALS, Natural Resources Canada, Hamilton, ON L8P 0A5, Canada; Frank.Czerwinski@nrcan-rncan.gc.ca

**Keywords:** automotive lightweighting, circular economy, sustainability, lightweight alloys

## Abstract

The automotive lightweighting trends, being driven by sustainability, cost, and performance, that create the enormous demand for lightweight materials and design concepts, are assessed as a part of the circular economy solutions in modern mobility and transportation. The current strategies that aim beyond the basic weight reduction and cover also the structural efficiency as well as the economic and environmental impact are explained with an essence of guidelines for materials selection with an eco-friendly approach, substitution rules, and a paradigm of the multi-material design. Particular attention is paid to the metallic alloys sector and progress in global R&D activities that cover the “lightweight steel”, conventional aluminum, and magnesium alloys, together with well-established technologies of components manufacturing and future-oriented solutions, and with both adjusting to a transition from internal combustion engines to electric vehicles. Moreover, opportunities and challenges that the lightweighting creates are discussed with strategies of achieving its goals through structural engineering, including the metal-matrix composites, laminates, sandwich structures, and bionic-inspired archetypes. The profound role of the aerospace and car-racing industries is emphasized as the key drivers of lightweighting in mainstream automotive vehicles.

## 1. Introduction

Lightweighting is becoming the major trend, reaching many industrial sectors associated not only with all forms of transportation but more broadly with civil infrastructure, manufacturing, and clean energy technologies [1]. In contrast to common perception, the lightweighting objectives are not exclusively focused on the reduction of weight but cover also other aspects involving the structural efficiency as well as the economic and environmental impact. In industry, reducing the weight of a product not only consumes fewer resources for its manufacturing but also requires less energy for its transportation, thus preserving natural resources and reducing the harmful pollution. Although lightweighting is not a new concept and aerospace has been on the lightweight path since its origin, while other sectors have also pursued it for decades, it is re-emerging as the mature, enormous growth course that is driven by sustainability, cost, and performance.

The core lightweighting objectives can be achieved through a number of individual strategies or their combinations that balance the design and material factors. The aim of lightweight design is to build structures with a minimal use of materials and an optimized utilization of the material strength, with numerical methods being developed to model the complex geometries of lightweight structures, e.g., in a parametric isogeometric environment [2]. The material selection has many aspects and just increasing its strength alone leads to a design weight reduction without changing its specific density. Through exploring this factor and using the high strength, Nb-containing weathering steel for, currently, the tallest bridge in the world, Viaduct de Millau, France, allowed for the reduction of its overall weight by 60% and for the related carbon footprint through fabrication, welding, construction, and transportation [3].

The ultimate lightweighting goal can be accomplished, however, through an application of lightweight materials and by combining their unique features with other strategies [4]. An increasing demand for lightweight materials led to an expansion of research towards novel solutions with strategies for achieving lightweighting goals through structural engineering, including metal-matrix composites, laminates, sandwich structures, and bionic-inspired archetypes. This report provides an overview of the current lightweighting strategies and materials, with a major focus on structural metallic alloys and their present and possible future applications in automotive transportation. It is a general statement, expressed by global automakers, that the vehicle weight reduction is a core part of the overall technology strategy that the industry will utilize to achieve the future targets of energy consumption, emissions, safety, and affordability.

## 2. Lightweighting as a Part of the Circular Economy

**Figure 1:**
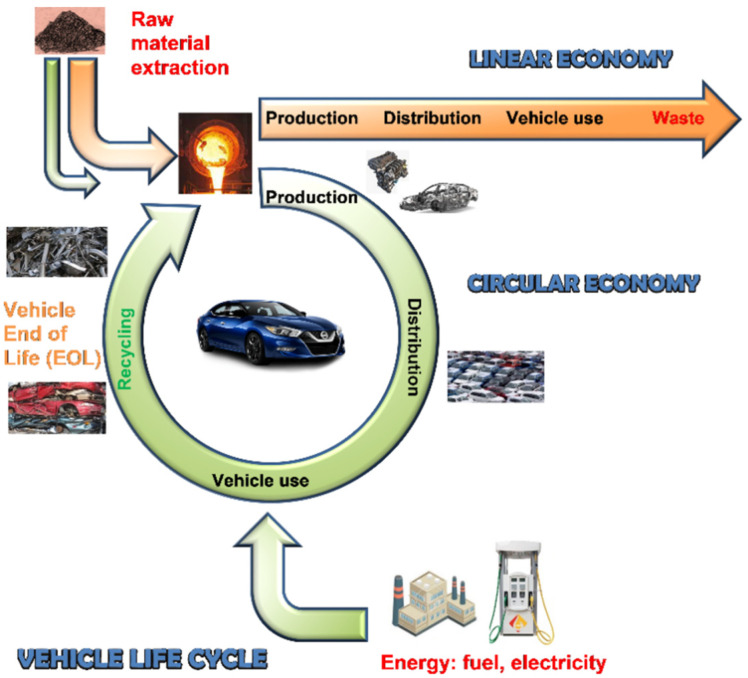
Schematics explaining the vehicle Life Cycle Assessment that encompasses all phases of the product cycle, from raw material extraction to end-of-life recycling and disposal.

In contrast to the *linear economy,* with its predisposition towards wasting valuable resources, the concept of the *circular economy* offers opportunities for a more productive use of materials through recirculating their larger share through reuse and recycling, thus reducing waste in production and extending the lifetimes of products, and through associated policies (Figure 1). The purpose of moving towards a circular economy is to slow down the depletion of scarce natural resources, reduce environmental damage from an extraction of raw materials, and reduce pollution caused by their processing, use, and end-of-life (EOL) recycling of materials [5].

Recirculating materials drastically increases their abatement potential and cuts CO_2_ emissions, since producing secondary materials through recycling results in far lower emissions than producing primary materials. According to [6], current EOL recycling rates for only eighteen metals, including silver, aluminum, gold, chromium, copper, iron, manganese, niobium, nickel, lead, palladium, platinum, rhenium, rhodium, tin, titanium, and zinc, out of the sixty considered are above 50%. For specialty metals, such as scandium and yttrium, as well as the rare earth elements, EOL recycling rates are 1%. In contrast, fully recyclable materials, such as aluminum with more than 90% of its content included in a car being recycled, perfectly suits the circular economy.

To investigate the environmental impact of products and processes during their entire life cycle, a methodology of the Life Cycle Assessment (LCA) has been increasingly used [7]. For a transportation vehicle, the assessment considers the impacts from the manufacturing stage, with material production and vehicle assembly; through to the use stage, with fuel production and combustion; and to the EOL stage, with final disposal and recycling. This is valid for electric vehicles: although they produce just one-third of the lifetime emissions of internal combustion engine cars, electric vehicles maintain the CO_2_ footprint created in the mining, manufacturing, shipping, and recycling of the vehicle components.

Different materials have different potentials in reducing the weight of vehicles and different carbon footprints for production and recycling. For some metals, their energy-demanding manufacturing process affects the potential energy savings during their service in the vehicle. This is particularly valid for aluminum and magnesium, known by their large energy input required during the primary metal production.

## 3. Lightweighting as a Solution to Sustainable Transportation

The automotive industry is going through an accelerated transformation with advancements in innovative technologies and changing of consumer preferences. Its business model that was valid for the last century to design, manufacture, sell, and service vehicles is anticipated to experience radical change through the involvement of intelligent mobility technologies. Such directions of sustainability, technology advancements, electrification, autonomous driving, consumer expectations, and personal mobility will drive changes over the next decade. It is predicted that the future transportation will be dominated by autonomous, connected, electric, and shared (ACES) vehicles that will alter the way consumers interact with vehicles. A lightweight vehicle is seen by automakers as a core aspect for sustainable mobility, improving both the fuel consumption and CO_2_ emissions.

An inclusion of the term *mobility* into recent literature may cause some terminology confusion by creating an impression that it aims to sound superior to the existing term *transportation* [8]. How do we define both terms? According to the dictionary definition, a clear difference exists between transportation, describing the process of moving goods or people, and *mobility,* describing the ability to move or to be moved. In support of mobility, it is argued that it offers a holistic approach that understands both the people and cargo transportation, with an emphasis on non-motorized means of transportation. In contrast to transportation, mobility is not limited to the existing infrastructure and behavior patterns of the market; it affords opportunities in unique ways. There are examples where both terms are used together as mobility and transportation, understood as the movement of goods and people, and the various means by which such movement is accomplished. According to some opinions, however, introducing the mobility term is solely rhetoric and a marketing strategy for making something look new and modern.

**Figure 2:**
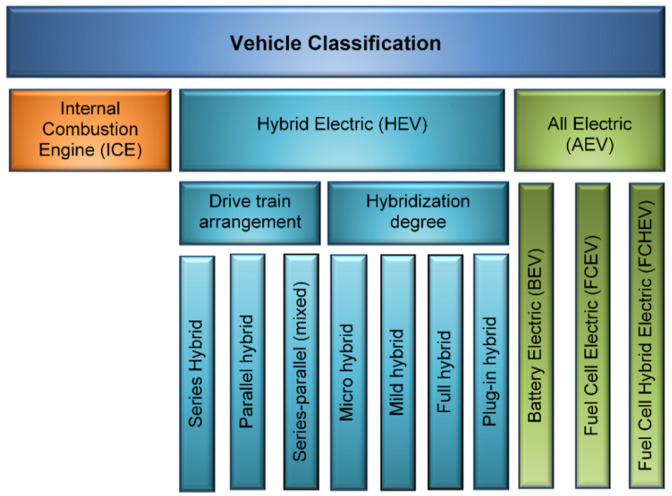
Classification of transport vehicles, showing current types of hybrid and electric solutions.

Four main areas are anticipated to dominate the automotive technology development in the near future; more efficient internal combustion engines, electrification/energy storage, lightweight structures, and both powertrains and power electronics [9]. The current development activities are heavily oriented towards the electric vehicle, which has improved energy efficiency with respect to the internal combustion engine vehicles [10] and their commercial market evolutions are outlined in [11,12] (Figure 2). At the same time, new challenges are created that include, for example, energy storage systems and power conversion topologies for electric vehicles. Commonly available energy storage devices in electric vehicles are fuel cells, batteries, ultra-capacitors, flywheel, and photovoltaic arrays that have to achieve high power and energy density to decrease the charging time [13]. Currently, the main sources of energy for electric vehicles on the market are batteries with fuel cells, while photovoltaic cells are used rather as an auxiliary power supply. A number of hybrid vehicles use supercapacitors.

According to the International Organization of Motor Vehicle Manufacturers, 91.8 million vehicles were produced in 2019. It is predicted that the automotive lightweight materials market will reach USD 99.3 billion by 2025 after continuous growth of the compound annual growth rate (CAGR) by 7.3% from USD 69.7 billion in 2020 [14]. In this market, metals are anticipated to remain the dominant material choice, constituting its largest segment. 

### 3.1. Internal Combustion Engine (ICE) Vehicles 

**Figure 3:**
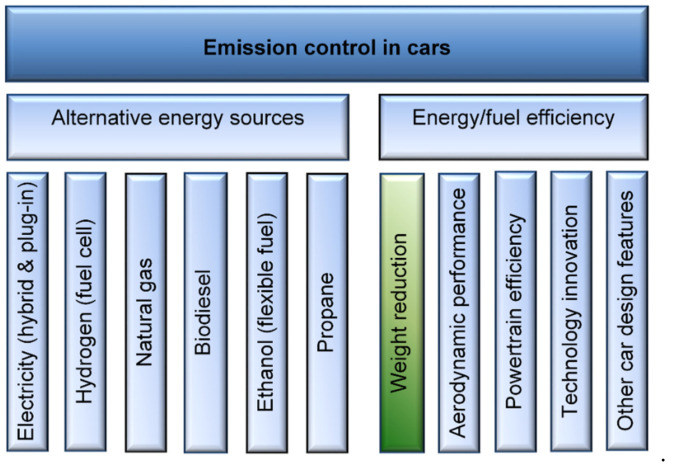
Major factors affecting the GHG emission in automotive vehicles and the vehicle weight reduction as the contributor.

For a transportation vehicle, the utmost goal is the reduction of the use-phase energy demand while retaining or improving the vehicle performance. For the ICE vehicles, improvements include the direct injection, friction reduction and turbocharging, higher gear count transmissions, electrification of power steering, start–stop systems, and aerodynamics. As shown in Figure 3, the vehicle weight reduction is one of many the contributing factors to its performance and fuel/energy economy. 

For ICE, reducing the vehicle weight improves the fuel economy with typically cited rates of 10% less weight and 6–8% less fuel, or 100 kg of a weight reduction reduces the fuel use by 0.3 to 0.5 L/100 km, corresponding to a reduction of 8 to 11 g of CO_2_/km. Although the lightweight vehicles are superior in meeting the requirement for sustainable development, there is frequently a higher cost of lightweight materials involved. 

This cost can be offset in part by secondary mass savings, understood as mass reductions, that may be achieved in structural (load-bearing) vehicle parts when the gross vehicle mass (GVM) is reduced [15]. Mass decompounding is the process by which it is possible to identify further reductions when secondary mass savings result in further reduction of GVM. The lower vehicle weight requires smaller engines and suspension affects both the efficiencies and lower number of overall parts, which reduces tooling and assembly costs. Thus, the vehicle design determines the reduction of subsystems’ weight, which results from the overall vehicle weight reduction. Maximizing secondary mass savings (SMS) is a key tool for maximizing the vehicle fuel economy.

### 3.2. Electric Vehicles

Over 100 new models of battery electric vehicles (BEVs) were announced for release by the world-leading automakers by 2024, which will increase the EV sales to 30–35% among passenger vehicles by 2030 [16]. It is a common understanding that lightweighting will continue to be in focus even as the industry is shifting from ICE to EV [17].

**Figure 4:**
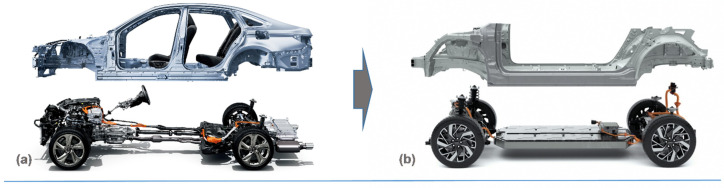
Architecture difference between internal combustion engine vehicles and battery electric vehicles: (**a**) ICE, Toyota Crown redesigned platform, 2018 [18], and (**b**) BEV, Hyundai/Kia/Genesis E-GMP platform, 2021 [19].

Electrification poses additional challenges since the batteries or added electrical components make them heavier than the conventional ICE vehicles. Since EVs are typically 125% heavier than ICE equivalents, there is a need to reduce weight in order to increase the driving range from a single battery charge. For electric vehicles, a 10% weight reduction typically equals a 13.7% increase in range. While the objective of lightweighting is similar in both ICE and EV, the differences in the product architecture affect the scope and approach for lightweighting in EV. As shown in Figure 4a,b, BEVs have a fundamentally different architecture than ICE vehicles, with large battery packs as the dominant feature rather than a large engine bay [18,19]. The need to secure a large, heavy battery pack at the bottom of the vehicle and the desire to use one platform for multiple vehicles will drive the automotive industry back into the design of frame arrangement for BEVs. 

Currently, electric vehicle manufacturers drive the demand for lightweight materials, with opportunities in the battery-carrier weight optimization, improved energy dense battery chemistries, and range management [20]. The powertrain of a full-battery BEV with a 35.8 kWh battery pack and 100 kW electric motor is nearly 125% heavier than a standard ICE vehicle powertrain. Moreover, lightweighting has a substantial impact on the cost of BEVs, with potential savings resulting from secondary weight reductions and downsizing of both the battery and drivetrain components, while keeping the same range [21]. Modelling of the estimated impact of lightweighting was found to be significant, totaling to about €4 per kg of mass reduction for a compact car and around €7 per kg for a high-range SUV, assuming a 400 km range. According to the Roadmap 2020 [22], weight reduction from the design optimization, material selection, and part-count reduction for ICE will reach 2–10% by 2025 and 20–25% by 2035, while for BEV it will be even higher, reaching 10–15% by 2025 and 20–30% by 2035. 

At the same time, there are views that the transition from ICE to EV changes both the goals and design considerations regarding lightweighting and how sensible lightweight construction measures are for electric cars [23]. According to this view, the heavier cars have higher kinetic energy at the same speed as that of lighter cars and can, therefore, recover more energy when recuperating during periods of slowing down. It should be kept in mind that after braking, vehicles usually accelerate again and the heavier vehicle must then use more energy to accelerate to the same speed as the lighter vehicle.

## 4. Design as the Lightweighting Strategy

The underlying idea of lightweight design is to create structures with the minimum weight while meeting the essential requirements of technical performance and safety. 

### 4.1. Lightweighting Design (LWD): Structural Optimization

**Figure 5:**
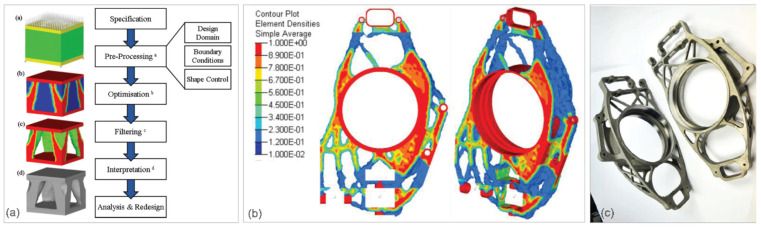
A concept of lightweight design through topology optimization: (**a**) topology optimization work-flow, with the example of a 75% mass reduction for a cube under a compressive load on the top face; (**b**) post-optimization result, showing element pseudo-densities of >0.25; and (**c**) structural member of the vehicle suspension assembly after EBM manufacturing and CNC machining [25].

The optimum lightweighting is achieved through an implementation of all lightweighting strategies that combine LWD with numerically optimized structures and advanced lightweight materials, where both the materials and structures are fabricated with effective manufacturing methods [24]. The structure design optimization involves the size, shape, and topology of the developed component and aims at distributing materials within a component to reduce its use as well as to enhance the structural performance, such as regarding higher strength and stiffness, better crashworthiness, and vibration performance. Topology optimization is a simulation-driven design technique used to create conceptual structures [25]. It is an incredibly powerful tool and can be used with the objective of mass reduction or thermal control. Since resultant topologies are often too complex for traditional processing, additive manufacturing seems to be the key solution (Figure 5a–c). 

Closely related to the part design is the so-called system lightweight design, understood as a combination of parts that is lighter than a reference system [26]. A weight reduction of the vehicle is achieved through the integration of multiple parts or functions into a single part or system to reduce the weight of an assembly. 

**Figure 6:**
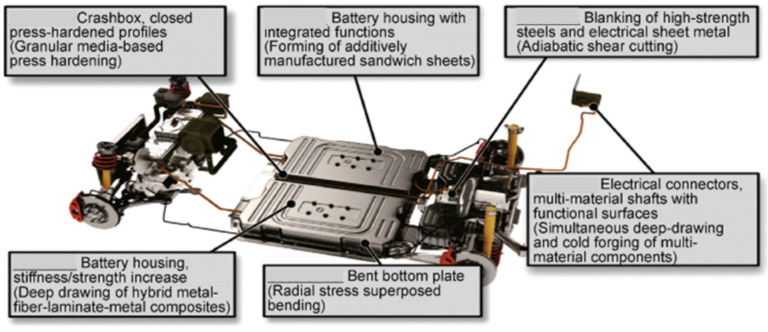
Examples of manufacturing processes that can be used for the lightweight components of electric vehicles [26].

The objectives of lightweight design are enhanced through innovative processes of components manufacturing. The development of advanced manufacturing technologies, such as additive manufacturing, advanced metal forming, joining, and thermomechanical treatments, not only enable the application of advanced materials but also relax constraints, enhancing the flexibility of multiscale structural optimization. Examples of processes that can be used to manufacture lightweight components of electric vehicles are shown in Figure 6. Such processes include innovative forming technologies of lightweight components, hot stamping of hollow profiles with high strength and high stiffness, and forming with hardening of semi-finished products manufactured by additive manufacturing. 

### 4.2. From a Material Substitution to Multi-Material Design (MMD)

In the past, automotive lightweighting was seen as a simple substitution of materials, mainly aluminum for steel. The present consensus is that the optimized multi-material design is the future direction. An introduction of multi-material structures with highly integrated light metal applications, using the material with the best properties for the given requirements in the right position, is required for modern transportation vehicles [27].

Multi-material design is considered as one of the strategies that allows for achieving product efficiency and represents the current forefront of the lightweight design trend in automotive manufacturing. A combination of different materials can be used to improve both the components’ properties, manufacturability, and the end-of-life recyclability. It is commonly accepted that implementing many materials with different characteristics during a design leads to the higher product performance in terms of its functionality, manufacturability, cost, and aesthetics.

It should be emphasized that the multi-material design may refer not only to the entire vehicle but also to the individual vehicle components. To describe them, terms of smart or hybrid components are used, where the component essential properties, such as strength, ductility, or crashworthiness, would change in different areas as per design requirements. The smart/hybrid components often consist of two or more material combinations, such as metallic alloy–carbon-fiber compounds, in order to fully exploit the property advantages of different materials. 

**Figure 7:**
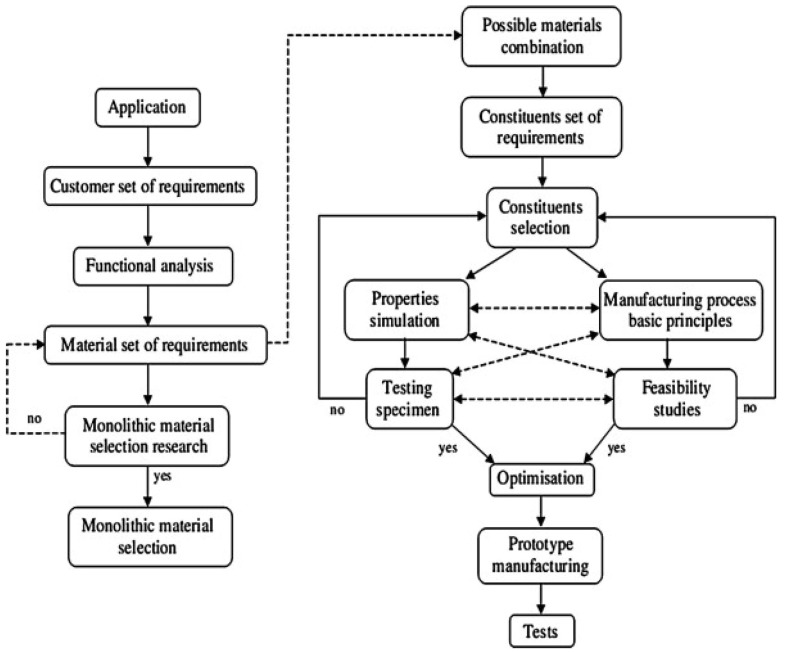
An example of the multi-material selection algorithm for lightweight design, taking into account product recyclability [29].

The design of multi-material vehicle components leads to various design options and trade-offs between conflicting development goals, making the development of suitable lightweight solutions for large-scale production very complex. As a result, a number of solutions are proposed in the literature. The methodological approach of developing multi-material components, which combines characteristics property modelling (CPM) and property-driven development (PDD) with extended mapping matrices (EMM), serves as a method to identify strategies to design concepts, as proposed in [28]. The method that is capable of guiding designers in multi-material selection for lightweight designs, taking into account product recyclability, is portrayed in Figure 7, where a multi-objective genetic algorithm is utilized to approximate the set of optimal solutions in material selection while incorporating recyclability [29,30]. To solve the compliance–minimization problems and apply it to both automotive concepts and lightweight design, algorithms for multi-material topology optimization were developed [31]. They show that for the same weight, the optimum designs achieved by the multi-material topology optimization method were much stiffer than those achieved by the standard single-material topology optimization. 

### 4.3. Multi-Material Lightweight Vehicles

The multi-material lightweight ICE vehicle was developed by Magna International and Ford Motor Company as a result of a US Department of Energy project [32,33]. A car equivalent to a five-passenger sedan (for Fusion 2013, full vehicle mass is 1559 kg), designed using the commercially available materials and production processes, achieved 1195 kg (23.5% savings), enabling the application of a 1.0 L three-cylinder engine. This resulted in total life cycle mass-induced fuel savings of 3642 L (or 962 gallons) and a projected combined cycle fuel economy of 34 mpg (6.9 L/100 km) compared to 28 mpg (8.4 L/100 km) for the 2013 Ford Fusion. In the Mach II design, which used materials and processes that have some initial research but are not ready for high volume production, achieved the total weight of 761 kg (51.1% reduction) [34].

**Figure 8:**
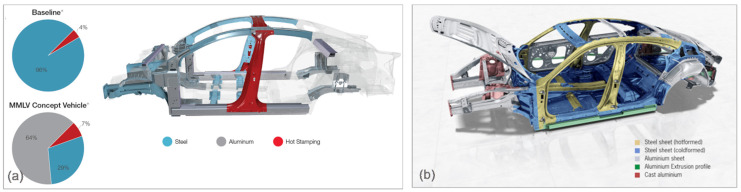
Multi-material designs of ICE and EV (body-in-white): (**a**) ICE, multi-material lightweight vehicle (MMLV), Magna, 2015 [33], and (**b**) EV, Porsche 800 V Taycan electric sports car, 2019 [35].

A different solution is presented for the Porsche 800V electric sports car, Taycan, with an optimized mixture of materials for maximum strength [35]. The fully galvanized body is a mix of aluminum and steel as the main materials. The strut mounts, axle mounts, and rear side members are made of die-cast aluminum and the shock absorber mounts are made of forged aluminum. The front side members combine an aluminum shell construction with extruded sections. Thus, apart from the front and rear-end components, the complete outer skin is made from aluminum, with its total content of around 37%. The difference between both designs can be deduced from a comparison in Figure 8a,b.

## 5. Ferrous Alloys as a Part of the Lightweighting Strategy

Ferrous metals, mainly steel, maintain the majority share of the automotive market, with about 70% of an average car weight consisting of steel sheet metal, forged steel parts, and cast iron. The ferrous alloys, due to their high density of about 7.8 g/cm^3^, are not generally classified as lightweight materials. However, there are efforts to modify the characteristics that make them effective in reducing the weight of transport vehicles. Increasing the strength alone leads to the design weight lowering without changing the specific density of materials, while applying high-strength steels lead to thinner sheets, resulting in a vehicle weight reduction. As a parallel approach, there are continued efforts regarding an incremental density reduction of ferrous alloys, including steel.

Steel, when accompanied by advanced manufacturing processes and innovative design concepts, is seen as the environmentally optimal and affordable material for transport vehicles. The attractive features of steel include low cost, manufacturability, recyclability, and the availability of specialized alloys. In terms of sustainability, the steel recycling process is considered simpler than that of aluminum in part due to the magnetic properties allowing for scrapyard sorting [36]. All steel grades can be melted together and remixed to produce new compositions. The steel recycling results in environmental benefits and reductions in CO_2_ emissions; production of 1 t of steel through the electric arc furnace route consumes only 9–12.5 GJ/t of crude steel, whereas the blast furnace steel consumes 28–31 GJ/t of crude steel, consequently representing enormous energy savings [36]. In contrast, aluminum is more expensive to recycle, requiring the different grades to be separated before melting to preserve the grades’ quality.

### 5.1. Reducing the Density of Steel

The term “low-density steel” was introduced in the 1930s when referring to the Fe–Mn–Al–C system [37]. Density reduction attempts were continued in the 1950s with research on the replacement of costly Ni and Cr in stainless steels with cheaper Mn and Al, respectively. As a next step, Fe-based aluminides (FeAl and Fe_3_Al) were researched as less expensive replacements for stainless steels and Ni-based superalloys [38].

**Figure 9:**
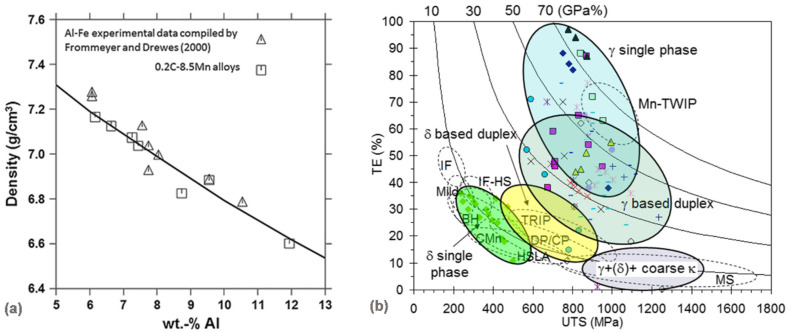
Reducing density of steels for automotive applications: (**a**) density as a function of aluminum content for binary Fe-Al18 and for quaternary 0.2C−8.5Mn (wt.%) alloys, wherein the continuous line corresponds to the density calculation for the quaternary system using Thermo-Calc [39]; (**b**) elongation (TE) as a function of ultimate tensile strength (UTS) in Fe–Mn–Al–C alloys (solution-treated and water-quenched strips) [40].

To reduce the steel density, aluminum is used as an essential alloying element. The addition of Al to Fe–C steels leads to a reduction in both density and in the Young’s modulus. A 1.3% reduction in density and a 2% reduction in Young’s modulus are obtained per 1 wt% addition of Al [39]. As a result, the density reductions of 8–12% are obtained in comparison with low-carbon steels (Figure 9a). A combination of properties and density reductions shows potentials for automotive applications. However, the aluminum alloying contributions required for lightweighting also introduce detrimental changes to the steel microstructure.

Fe–Mn–Al–C steels, previously developed in the 1950s for replacing Fe–Cr–Ni steels, are currently generating great interest regarding potential applications for structural parts in the automotive industry because they are lighter [40,41]. With increasing aluminum content, however, brittle intermetallic compounds can form, leading to poor ductility. It was showed [42] that an FeAl-type brittle and hard intermetallic compound (B2) can effectively be used as a strengthening second phase in high-aluminum and low-density steel while alleviating its harmful effect on ductility by controlling its morphology and dispersion, as depicted in Figure 9b.

### 5.2. Increasing the Strength of Steel: Advanced High-Strength Steels (AHSS) 

According to the strength criterion, steels for the automotive industry are classified as traditional mild steel, conventional high-strength low alloy (HSLA) steel, and advanced high-strength steel (AHSS) [43]. Due to constant development, there is no consistent global terminology to classify high-strength steels. The AHSS steels have complex multiphase microstructures containing bainite, martensite, and residual austenite, which allow them to achieve unique mechanical properties. Typically, the AHSS has the ultimate tensile strength (UTS) between 450 and 800 MPa. The steels with an UTS exceeding 1000 MPs are described as ultra-high-strength steels. This is separate from the metallurgical designation that is based on the steel composition, processing, and microstructure.

**Figure 10:**
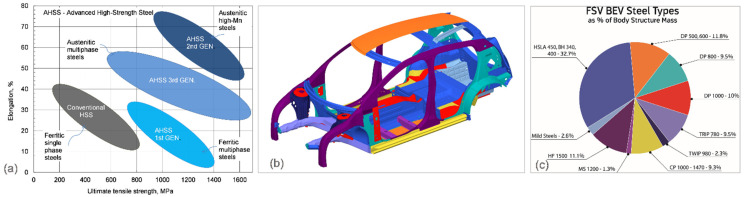
AHSS steels in electric vehicles: (**a**) schematics of formability–strength relationships in AHSS and (**b**,**c**) contribution of various steel grades to battery electric vehicles developed within the Future Steel Vehicle (FSV) program with 95% HSLA and AHSS, and with 48% having a strength over 1000 MPa, according to the World Steel Association [46].

During the last five decades, as an extension of HSLA steels, three generations of AHSS were developed for the purpose of lightweighting in the automotive industry (Figure 10a). Depending on the steel generation, there may be some challenges regarding formability and weldability [44]. The first generation of the AHSS family includes dual phase (DP), complex-phase (CP), martensitic (MS), and regular transformation-induced plasticity (TRIP). The second generation of AHSS includes a new generation of transformation-induced plasticity (TRIP), hot-formed (HF), and twinning-induced plasticity (TWIP) steels. Both the first and second generation of AHSS are designed to meet the functional performance demands of certain parts of the automotive vehicle. In recent years, new AHSS grades have been developed, for example, Extra-advanced High-strength Steels (X-AHSS) and Ultra-advanced High-strength Steels (U-AHSS), as well as various types of the so-called third-generation AHSS steels, e.g., TRIP-aided bainitic ferrite (TBF) and Quenching & Partitioning (Q&P), or different types of NanoSteels, all these with the primary aim to provide even higher strengths with significantly increased formability [45].

Being stronger than the conventional automotive steel, AHSS allows for thinner sheets than the conventional sheets, which enables automakers to build vehicles that are both strong and lightweight. The AHSS grades are anticipated to dominate the vehicles material share in the future, especially in body-in-white applications (Figure 10b,c) [46]. With AHSS, automotive manufacturers can reduce the weight of a vehicle body by as much as 25%. According to the Steel Market Development Institute, with an addition of the third generation of AHSS, that weight reduction can reach 30–40%.

## 6. Conventional Lightweight Alloys with Their Current Limitations

Lightweight materials are the essence of the weight reduction strategy and their typical list includes aluminum, magnesium, beryllium, titanium, titanium aluminides, structural ceramics, and composites with polymer, metal, and ceramic matrices [4]. Currently, only aluminum and magnesium alloys are of commercial interest for automotive manufacturers.

### 6.1. Aluminum

**Figure 11:**
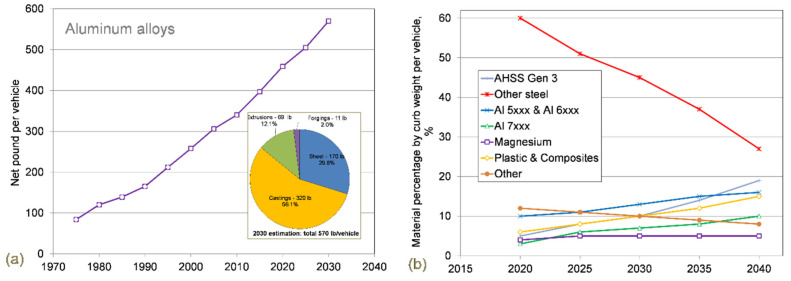
Current and predicted contribution of major automotive materials: (**a**) aluminum content growth in North American vehicles based on data from [47] and (**b**) average vehicle structure (body-in-white and closures) material percentage by curb weight per vehicle based on data from [49].

Aluminum offers a lower-weight alternative to steel and it fits greatly into a circular economy since it is highly recovered and reused in new products. The current North American Light Vehicle Aluminum Content and Outlook report reveals that aluminum is the fastest growing automotive material (Figure 11). Since 2010, aluminum usage in the automotive industry has grown from 154 kg (340 lbs) per vehicle to 208 kg (459 lbs) per vehicle in 2020 [47]. It is expected to grow further to 233 kg (514 lbs) per vehicle by 2026, up 12% from 2020 levels. According to European data [48] the amount of aluminum increased from 50 kg per vehicle in 1990 to 151 kg currently, with a projection of 196 kg per vehicle by 2025. However, an application of aluminum is still limited mainly to the engine, transmission, wheels, heat exchangers, chassis, and suspension. Currently, cost is seen as the main barrier to increased aluminum use. 

#### 6.1.1. Technology of Components Manufacturing: Castings and Sheet

The major technology of manufacturing the automotive components from aluminum is casting (Figure 11a inset). High-pressure die casting (HPDC) offers benefits compared to other production technologies, such as chill casting or sand casting, through significantly lower production times for individual parts while ensuring high repeatability, a broader range of shapes, and lower individual cost. The predicted contribution of aluminum, along with steel, magnesium, and other materials, to vehicles up to 2040 is shown in Figure 11b [49].

The development history of aluminum sheets for the automotive industry covers Al–Mg, Al–Cu, and Al–Mg–Si within 2000, 5000, and 6000 series [50]. Recently, heat-treated 6000 series alloys attracted attention for skin panels, as the 5000 series is prone to stretch-stain marks, and has the added value of becoming stronger after being processed through a paint-curing cycle, increasing the exterior dent resistance with a focus on dissimilar materials-joining technology for aluminum and high-strength steel [51,52]. The auto industry is looking for higher-strength aluminum materials needed for strength-driven safety-critical parts and experimental 7000 alloys are developed for these applications [53]. Further expansion of aluminum use can be achieved through an application of sheets for automotive hoods, trunk lids, outer panels such as doors, and protection covers including heat insulators.

**Figure 12:**
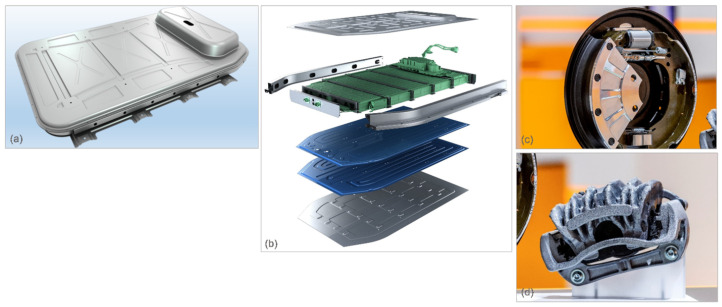
Considered application of aluminum in electric cars: (**a**,**b**) aluminum sheet battery enclosures of 1st and 2nd generation by Novelis [54] and (**c**,**d**) concept of lightweight drum brake and bionic-inspired caliper by Continental [55].

The novel application example of aluminum in electric vehicles concerns the aluminum sheet battery enclosure that can help extend the driving range, allowing vehicles to travel up to 10% further on a single charge [54]. The enclosure accommodates all battery cell types and is designed particularly for BEV, with larger power packs such as pick-up trucks, sport utility vehicles, and crossovers. The enclosure is built using Novelis Advanz™ aluminum alloy and shows advantages when compared to aluminum extrusion and casting-intense designs. Moreover, it is up to 50% lighter than an equivalent steel design and is well suited for the mass production of vehicles (Figure 12a). The 2nd generation enclosure was developed specifically for automakers to use the advanced CTP (cell-to-pack) battery packaging architecture, which is 15% to 20% more compact than traditional cell configurations, requires fewer parts to build, has lower cost and increases the volumetric energy density (Figure 12b).

Aluminum is also considered for EV brakes, where regenerative braking sends back most of the car energy into its battery rather than dissipating it in the form of heat from the friction of a conventional braking system [55]. The aluminum brakes can handle EV-reduced braking demands with less cost and weight than the alternative massive still brakes. The concept consists of a light aluminum wheel combined with the bionic lightweight caliper, which is proposed to also use an aluminum rotor, and the intelligent hydraulic drum brake with a lightweight composite drum. Another advantage of drum brakes is that the drum seals the braking components inside (Figure 12c,d). When the enclosing drum is aluminum, there is no rust or corrosion to detract from the vehicle appearance.

#### 6.1.2. Semisolid Casting

To improve the part quality and simultaneously preserve the low cost rate typical for casting, semisolid processing technologies were developed. Instead of the fully liquid alloy, it explores a semisolid slurry where a solid fraction has a spheroidal morphology as a result of liquid-metal engineering. Such a slurry has thixotropic properties and therefore flows during die filling in a more laminar mode, forming low porosity (high integrity) parts after solidification and having better properties. During heat treatment, components do not experience blistering, as frequently seen in conventional castings. Between two essential routes of the semisolid processing, namely thixocasting and rheocasting, the latter is more developed commercially [56]. It is of interest that semisolid routes can often operation for wrought alloys as well. The Swirled Equilibrium Enthalpy Device (SEED) is one of several routes, available commercially, that starts from liquid alloy and produces high solid-fraction feedstock for semi-solid casting [57,58].The principle is based on achieving a rapid and controlled thermal equilibrium between a metallic crucible and the bulk of the molten aluminum via the selection of simple process parameters such as pouring temperature and stirring duration. Shot weights can reach up to 54 kg and can be readily adjusted. The SEED process can be retrofitted to both horizontal and vertical die casting or squeeze casting machines.

#### 6.1.3. Alloy Development Challenges

For cast aluminum alloys with an application in and around the combustion engine, its thermal stability, determining the material ability of retaining its properties-required temperatures over extended service times, is of key importance [59]. Currently, extending thermal stability to higher temperatures is the technology and knowledge barrier that prevents the substantial expansion of the application scope of aluminum alloys in the automotive industry. To overcome this barrier, alloying of aluminum with high melting-point transition metals [60] or rare earths [61,62,63,64,65] has been investigated.

#### 6.1.4. Automotive Life Cycle Assessment Model

**Figure 13:**
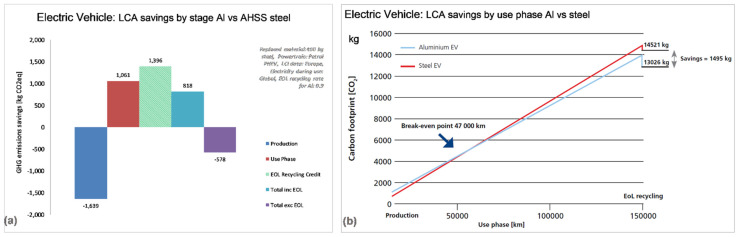
Example of Life Cycle Assessments of AHSS steel and aluminum in electric cars: (**a**) savings by stage and (**b**) savings by use phase. Details of the model, developed by the European Aluminum, are available from [66].

Aluminum is considered a preferred material for electric vehicles, as it offers value in terms of the manufacturing costs, weight savings, and performance, with cooling solutions, battery frames, and cables now routinely fabricated from aluminum [47]. In electric vehicles, the heat transfer capacity is particularly valid because the motor and batteries naturally develop large amounts of heat. Therefore, aluminum shields designed around the motor or batteries allow for efficient transfer of the heat generated, preventing overheating [16]. According to aluminum promoters [16], for the same crash performances, lightweighting through aluminum reduces both the production and operating costs of electric vehicles since a lighter car needs fewer batteries and less electricity to travel the same distance. The automotive Life Cycle Assessment model, developed following the ISO 14,040 framework, allows for calculating the impacts of selecting different materials for the purpose of the weight reduction of typical passenger cars [66,67]. One should remember that such an assessment tool is based on many assumptions. According to this model, aluminum provides CO_2_ emission savings in both ICE and EV, with the aluminum electric vehicle emitting 1.5 tons less of greenhouse gases over its complete life cycle (Figure 13a,b).

However, according to the steel promoters [68], while the aluminum-intensive vehicle may save some energy in the use phase, it would require approximately 30% more energy over its life than the vehicle made primarily from AHSS. This is caused by the eight-times higher energy required to produce a kilogram of primary aluminum metal in comparison to steel, associated with its more complicated extraction and refining process.

### 6.2. Magnesium

Due to its lightweighting characteristics, magnesium alloys have proven to be attractive structural materials for transport vehicles, as magnesium is 75% lighter than steel, 50% lighter than titanium, and 33% lighter than aluminum. Despite promising industrial applications at the beginning of the previous century by Volkswagen and Porsche, the projected usage of 50 kg per car in 2015 did not materialize and is still far away from reaching high-volume commercial applications [69]. This is in contrast to the research interest in magnesium. According to the statistical analysis of the literature data collected by Web of Science Core Collection, the growth rate of publications on magnesium alloy during 2008–2018 is significantly higher than the overall growth rate of alloy research papers. In the past 11 years, 21,440 papers on magnesium alloys were collected, reaching about 2000 papers per year, which accounts for 20% of all alloy research.

#### 6.2.1. Technology of Components Manufacturing: Castings and Sheet

Today, casting is the dominant manufacturing process for magnesium components, representing about 98% of all structural applications for magnesium [70]. This is due to the excellent castability of magnesium alloys, which exceeds other metals such as aluminum and copper. The unique solidification features of magnesium, including high fluidity and low susceptibility to hydrogen porosity, make it a good candidate for successful casting operations.

To expand the existing magnesium applications and to manufacture wrought components in commercially viable sizes with tight dimensional tolerances, adequate surface quality, and optimal mechanical properties, sheet metal-forming is required. Semi-finished products, such as strips and sheets, can further be transformed into net-shape final parts through a variety of manufacturing processes including laser or water jet cutting, stamping, bending, perforating, punching, incremental or press-brake forming, curling, roll forming, and spinning. The development of a low-cost magnesium sheet is thus an enabler to downstream processing. For example, in the automotive market, these parts could include hoods, trunks, inner door panels, and seat components, among other applications.

The obstacle to industrial-scale magnesium rolling is its inherently poor formability at room temperature related to the magnesium crystallographic structure. There are essential differences in the deformation and recrystallization mechanisms between magnesium and aluminum, and crystallographic texture engineering through a combination of intelligent processing through novel rolling techniques and alloying is seen as a possible solution [71,72,73]. There is also an expectation that the twin roll casting (TRC) technique could contribute to in the process. Twin roll continuous casting is seen as the technique that may overcome barriers of magnesium formability and allow for manufacturing sheet or strip products directly from a molten state. The technology takes advantage of the best features of both fundamental processes, namely casting and rolling, since it combines them into a single-step operation, thus reducing the manufacturing cycle time, energy consumption, pollutant emission, and final cost compared to traditional sheet production using a direct chill ingot casting [74].

#### 6.2.2. Semisolid Forming–Injection Molding

**Figure 14:**
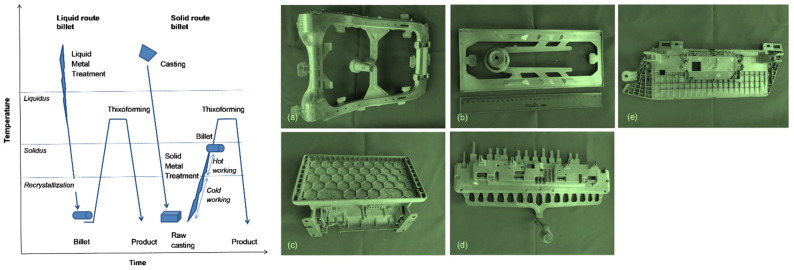
Schematics of semisolid processing and examples of automotive components manufactured from magnesium alloy using injection molding: (**a**) car seat backrest AM50, 1970 g; (**b**) car dashboard member AZ91D, 138 g; (**c**) car navigator member AZ91D, 280 g; (**d**) car dashboard member AZ91D, 710 g; and (**e**) car navigator member AZ91D, 278 g. Parts manufactured by SSD Magnesium, China [76].

In a search for novel technologies enabling the large-scale production of high performance, net-shape components from magnesium alloys, special attention is paid to the last three decades regarding semisolid processing. In contrast to aluminum, which favors rheocasting, magnesium semisolid processing is focused on routes that start from the solid alloy, called thixoformimg. The high affinity of magnesium to oxygen, its flammability while in the molted state, and the development of the processing route called semisolid injection molding (thixomolding) contributed to the popularity of magnesium injection molding [70]. The injection molding technology [75] represents a rather niche market and is used globally to produce automotive components, with examples shown in Figure 14. The components are generally of small and medium size with a large number of details difficult to obtain by conventional casting. Properties after injection molding are usually located between values of castings and wrought products; for cast alloys, the strength typically exceeds the values after casting, but for wrought alloys, the strength is generally below the values obtained in wrought products.

#### 6.2.3. Challenges with Automotive Applications

As shown earlier in Figure 11b, the predicted use of magnesium in automotive vehicles will remain at the low level of 4–5 kg per vehicle towards 2040. Examples of Mg applications include instrument panels, steering wheels, engine cradles, seats, transfer cases, and many different housing applications. In Europe, powertrain parts have been developed for the high-class platform with a use of 10 kg on average; in North America, interior parts in luxury cars use 15 kg on average; and in Korea and Japan, interior and powertrain parts in luxury sedans consume roughly 8 kg of magnesium [77].

Currently, there are still barriers to using magnesium in high-volume vehicle applications, as manufacturing and processing, in-service performance, and cost are seen as obstacles [78]. Quite often, magnesium is replaced from existing applications with a lower cost or better-performing material. In some cases, the use of magnesium parts is discontinued due to corrosion, creep, or other limitations of the magnesium alloy selected [79].

#### 6.2.4. Life Cycle Emissions

A large range of emissions from the primary magnesium production depends on its geographic location and the process type used to generate the primary metal is what makes it difficult to precisely determine the emission scale [80,81]. According to the Life Cycle Assessment of magnesium by the German Aerospace Centre (DLR) and an analysis of the entire life cycle of magnesium components for transport applications, the use of magnesium in transport applications lowers greenhouse gas emissions over the whole life cycle [82]. On average, magnesium shows higher emissions during component production compared to steel or aluminum on a per kg basis. These higher emissions are compensated during the use stage and the amount of saved fuel and emissions depend on the weight reduction. Magnesium components can save about 25% of their weight compared to aluminum. For calculating the overall difference to the reference component, the emissions of the overall life cycle results show a positive net balance of greenhouse gas emission for those magnesium production scenarios that represent the current magnesium market. It is also stated that general conclusions on the comparison of magnesium and aluminum parts cannot be drawn without ambiguity.

## 7. Lightweighting through Composites, Laminates, and Sandwich Structures

As has been proven in aerospace, composites have extensive capabilities in contribute to lightweighting. However, the application of a composite structure is generally driven by the trade-off between its lightweight performance and production costs. As a result, the wide use of advanced composites in the automotive industry is still thwarted by their high cost. For example, lightweight carbon-fiber composites weigh about one-fifth of that of steel but are as good or better in terms of stiffness and strength. However, the cost of carbon-fiber composites is at least 20 times as much as steel, which prevents their use in cars.

### 7.1. Reinforcing the Aluminum Matrix with Hard Discontinuous Particles

Metal-matrix composites (MMC), exploring the discontinuously reinforced aluminum matrix, have a potential for automotive applications due to their low density, good strength and ductility, and excellent thermal conductivity and corrosion resistance [83]. The reinforcement with solid lubricants, hard ceramic particles, short fibers, and whiskers is used to improve the aluminum low resistance to seizure and galling.

The emphasis has been on developing affordable aluminum MMCs reinforced with SiC and Al_2_O_3_ that reduce the weight and increase the engine efficiency, as well as replace other structural automotive components. Aluminum MMC with SiC can optimize high-performance automotive applications, including powertrain engine components, engine pistons, cylinders, connection rods, elements of vehicle braking systems (rotors), suspension systems, and chassis [84]. Newly developed hybrid composites with aluminum matrices have a significantly higher resistance to wear, higher specific stiffness, and higher resistance to fatigue [85].

**Figure 15:**
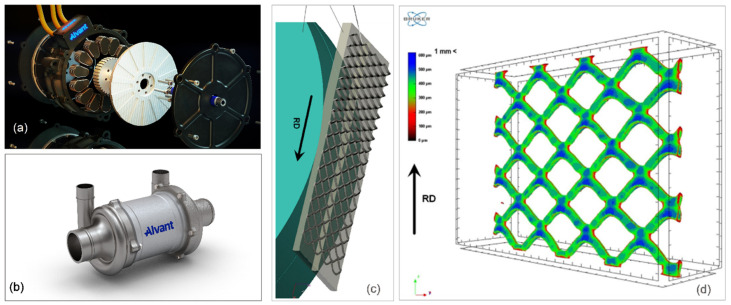
Aluminum matrix composite use in automotive parts: (**a**,**b**) MMC rotor of an axial flux electric motor and a hydrogen fuel cell compressor developed by Alvant, UK [86] and (**c**,**d**) composite manufactured by a reinforcement of the aluminum matrix through a steel mesh [87].

Adopting aluminum MMC with ceramic reinforcement for the rotor design to replace steel on the axial flux electric motor used in EV leads to 45–73% weight savings while increasing both the rotor power-to-inertia ratio potential and power density increase by 225–300% (Figure 15a,b) [86]. In modern EV, there is a need to optimize motor efficiency maps, for example, by improving the efficiency as a function of torque and speed, which ultimately determines the energy consumption of the vehicle.

### 7.2. Reinforcing the Aluminum Matrix with Steel Mesh

Sheet composite materials based on the aluminum matrix, reinforced with internal components such as an expanded steel mesh inlay, have great potential in the automotive industry. Although the steel reinforcement of aluminum sheets results in an increased density, it improves the strength and impact energy absorption in comparison with conventional aluminum alloys. An example of the reinforcement geometry is shown in Figure 15c,d. Roll bonding at increased temperatures of up to 500 °C was proven as the effective technique for manufacturing the steel composite [87]. Apparently, the mesh angle of the inlay of ferrite-pearlite steel was two times less sensible to the rolling reduction in comparison with a similar mesh of an austenitic steel. The maximal rise of the specific impact energy for 20% exhibits the reinforced composite rolled with a thickness reduction of 40% using a mill with smaller rolls. A similar reduction applied to the composite in the mill with greater rolls significantly deteriorated the impact properties. Twin roll casting was also found to be effective in manufacturing the AA1060 aluminum matrix reinforced with the 304 stainless steel wire mesh [88]. Since the process started from molten alloy, the mesh–matrix bonding took place during solidification in the semisolid state. In another attempt, stainless steel wire mesh was incorporated into the aluminum alloy matrix through hot processing in solid and mushy states and through the powder metallurgy route in the solid state [89].

### 7.3. Fiber Metal Laminates

**Figure 16:**
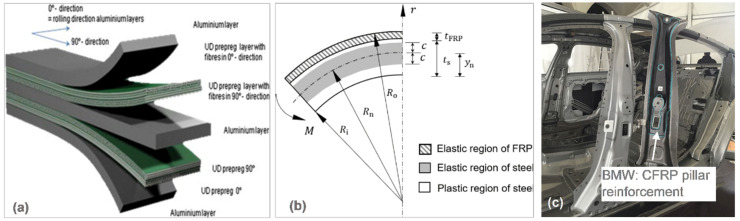
Application of fiber metal laminates in automotive parts: (**a**) general concept of FML [90]; (**b**) elastic and plastic regions of the steel–FRP composite under three-point bending [94]; and (**c**) 2016 BMW 7 Series (G12) CFRP B-pillar inner reinforcement [95].

Fiber metal laminates (FML), defined as a hybrid material consisting of alternating layers of monolithic metallic sheets and pre-impregnated fiber layers, take advantage of both metals and composites, and have shown great promise as lightweight structural materials in transport vehicles (Figure 16a) [90]. Originally developed to improve the fatigue resistance in aerospace applications, these laminates also exhibit other advantages, especially concerning the high strength-to-weight ratio [91]. As a result, FML may be thinner and lighter than aluminum alone. In the automotive industry, a floor assembly enabled by the development of FML stamp forming technology has shown a great potential of FML in providing comparable structural performance with a weight reduction of about 25% compared to a full-metal lightweight structure [92].

An automated, large-scale production process of lightweight car structures with a high stiffness-to-weight ratio was proposed through the combination of high-strength steel and carbon fiber reinforced plastics (CFRP) prepregs in a hybrid material/fiber metal laminate [93]. Carbon fiber reinforced plastics, a composite material made of carbon fiber that was impregnated with a thermosetting resin and formed by curing, is ten times stronger than steel but only one-fourth as heavy and combines the advantages of metal, such as strength and stiffness, with the ability to make lightweight products. The laminate can be further processed by forming technologies, such as deep drawing, and consists of two metal-sheet top layers with a CFRP core.

An application of fiber metal laminates in an automotive vehicle when preforming CFRP reinforcement resulted in an improvement in both the crashworthiness of the car body and the weight reduction [94]. The strengthened B-pillar assembly by rivet-bonding caused a weight reduction of 44%, while its crashworthiness was improved by 10% compared to that of the tailor-welded steel pillar (Figure 16b,c) [95].

Consider the example of a natural fiber metal laminate consisting of two layers of woven fabric of a tropical plant of the mallow family, namely kenaf, in the polypropylene matrix and with aluminum 5052-O as the skin of the car front hood, as described in [96]. The kevlar/basalt/aluminum AA8090 reinforced metal laminates were processed manually by compression molding and is proposed for high-strength commercial automotive applications, including in floorings, frames, and bonnets [97].

The three-layer laminate termed as Smart Steel^®^ was manufactured with the outer skins made of steel and the inner layer of a low-density, conductive, reinforced polymer core with a of density less than aluminum [98]. The lightweight laminate is produced as a coil and is a formable, weldable, and paintable substitute for monolithic, low-carbon steel, with up to a 35% weight savings at the same thickness. The laminate enables weight reduction while maintaining bending stiffness, an attribute that is critical in many areas of the vehicle.

### 7.4. Sandwich Structures

**Figure 17:**
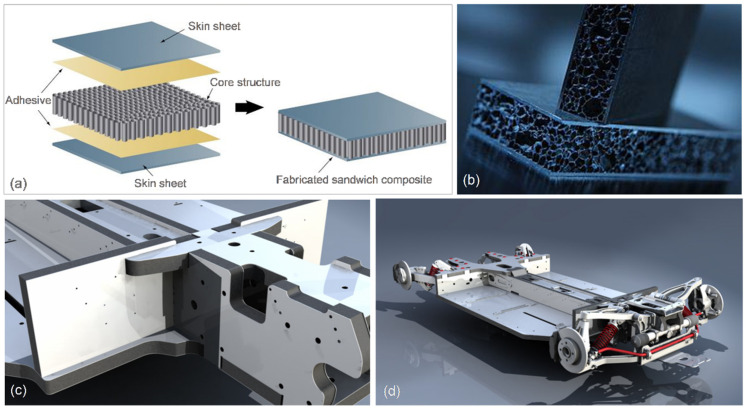
Sandwich-structured composites: (**a**) schematics showing elements of the sandwich structure [105]; (**b**) aluminum foam sandwich [105]; and (**c**,**d**) concept of applying the lightweight aluminum and ARPRO propylene-based foam sandwich material in a car (Inrekor, UK) [110].

A sandwich-structured composite consists of two thin high-strength skins that are separated by the thick and lightweight core [99,100,101]. The core material has typically a low strength but its higher thickness provides the sandwich structure with high structural stiffness, strength, and a high level of energy absorption potential, ensuring the overall low density. Sandwich structures are important innovative multifunctional solutions with advantages of low density and high performance (Figure 17a). The analysis performed using a methodology that combine the weight-optimization and technical cost modelling through an application-bound design cost revealed that sandwich structures are weight and cost-efficient in low to intermediate bending stiffness scenarios and torsional applications [102].

Sandwich composites belong to anisotropic materials, with their strength properties changing depending on the applied load direction. Since bending stresses reach maximum levels at the exterior, the material located further away from the centerline has a higher impact on bending strength and stiffness. Sandwich composites are becoming increasingly popular in the automotive sector and the challenge concerns how to replace the monolithic metals through this form without compromising the mechanical performance [103]. The classic methods of manufacturing and the assembly of lightweight sandwich structures involve many stages that make production expensive and require complex and sometimes difficult to use devices.

An aluminum foam sandwich (AFS) is comprised of a highly porous aluminum alloy foam core and two aluminum alloy face sheets (Figure 17b). The layers are firmly attached to each other by metallic bonding [104,105]. Various methods for making such foams are available. Some techniques start from specially prepared molten metals with adjusted viscosities. Such melts can be foamed by injecting gases or by adding gas-releasing blowing agents, which decompose in situ, causing the formation of bubbles [106]. Aluminum foam sandwich panels may be fabricated via liquid diffusion welding and glue adhesive methods [107]. Although the material is ready for high-volume production, the number of industrial applications is low because of its high costs, the lack of sufficient knowledge to use it during the vehicle design, and missing reference applications [108].

The example of a sandwich structure proposed in [109] consists of the SK5 (DIN 17350) steel skins, sheet molding compound (SMC) core, and two thin adhesive layers. The sheet molding core was made of 35 ± 5% chopped carbon fiber, with an average length of 25 mm, and the remaining volume fraction of vinyl-ester resin. The pre-cured SMC is normally manufactured in layers of variable thickness of 2–3 mm, which must be compressed and cured in order to obtain a stiff structure.

To further reduce the specific weight, ARPRO, a propylene-based foam, sandwiched between two thin sheets of aluminum, was developed and its proposed application for the entire automotive sub-frame (Inrekor, UK) is shown in Figure 17c,d [110]. The sandwich structure is light yet strong and cheaper than carbon fiber and can be completely recycled. The use of the material could reduce a car weight by up to 30%, corresponding to about 500 kg per vehicle.

## 8. Lightweight Structures through Bionic-Inspired Designs and Additive Manufacturing

Biomimicry, which include innovations inspired by nature, explores the strategies found in nature and adapts these biological models, systems, and elements to solve engineering problems. Bio-inspired concepts are increasingly used to design both vehicles and the materials used for vehicles.

### 8.1. Cellular Materials

Cellular materials are a special class of lightweight materials that are found in nature but are also increasingly used in technological applications [111]. They have complex structures accompanied by high-energy absorption, excellent damping properties, and formability useful for crash protection, thermal, and acoustic insulation properties.

Honeycomb structures, inspired from bee honeycombs, are attractive candidates to be widely used in lightweight automotive designs due to their high stiffness-to-weight ratio, high strength-to-weight ratio, cost efficiency, multi-functionality, and extraordinary energy absorption capacity.

**Figure 18:**
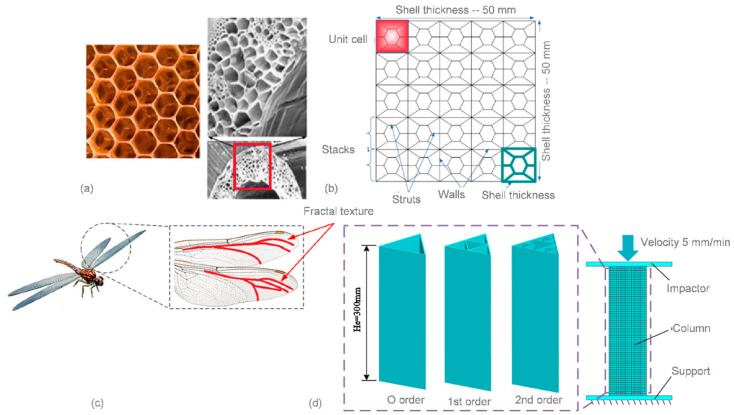
Bionic-inspired cellular structure design: (**a**) bee honeycomb pattern; (**b**) geometry of the cellular structure designed for numerical modelling [112]; (**c**) fractal texture of the wings of dragonflies; and (**d**) finite element model of the novel fractal structures [114].

The honeycomb-inspired cellular structure for electric vehicle battery protection during collisions was designed this way [112]. During numerical analysis, four different ways of applying the shell thickness, which affects the collapse behavior and performance metrics of the cellular structure, were examined (Figure 18a,b). Another bio-inspired lightweight sandwich structure was designed based on the microstructure of the cross-section of the beetle elytra. The traditional lightweight honeycomb sandwich structure was used for comparison with the new structure. Samples of the two structures were manufactured by 3D printing technology [113].

In another example, a bio-inspired design strategy for aluminum alloy thin-walled structures was proposed to improve the performance of out-of-plane crashworthiness by altering the material distribution [114]. In this process, a novel fractal thin-walled triangle column (FTTC) was designed and composed by iteratively applying the affine transformation of a base triangle up to second order. The fractal order has a major influence on performance. In particular, the SEA of the second-order FTTC is 89.6% higher than that of the zeroth-order (Figure 18c,d).

Cellular foams represent a bio-inspired material that has received increased attention for vehicle crashworthiness due to their lightweighting and excellent energy absorption capabilities that allow for significant weight reductions without compromising the structural safety aspects. It was found that the cellular foams, when used as a filler material in thin-walled energy absorbers, improve the crashworthiness performance through favorable changing of the deformation mode [115].

### 8.2. Additive Manufacturing for Complex Ultralight Structures

Manufacturability is an important limitation when designing lightweight structures. Advances in manufacturing technologies have led to the development of a new approach to material selection, in which complex designs can be created to achieve a specific mechanical objective. The new opportunities in the design of ultralight components are provided by additive manufacturing (3D printing), which allows for alternative, radical, and new design approaches for component internal structures. AM technologies make it possible to fabricate parts of any complexity [116]. Therefore, it is possible to redesign lightweight, geometrically complex parts without considering manufacturing constraints. The present limitation of AM concerns the range of alloys that it is able to print, as typical alloys used in industry are not adaptable for AM due to issues with the structure and properties. Therefore, developing novel metallic materials for AM is the main challenge for researchers looking at other processing techniques such as casting [117].

**Figure 19:**
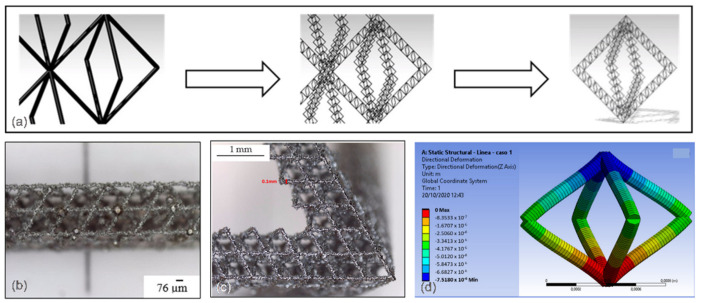
Additive manufacturing of ultralight components: (**a**) octahedron cell lattice of the structure; (**b**,**c**) detailed image of the structure manufactured by laser powder bed fusion using Inconel 718; and (**d**) deformation map during the vertical compression [118].

The idea of reducing weight in high-strength elements using replicative structures is shown in Figure 19a–d using laser powder bed fusion (L-PBF) as the basic manufacturing technology [118]. The idea is based on replicable basic structures in different orders of magnitude. The proposed structures are very light, in which the strength–weight ratio is brought to extreme limits. The final structure is a three-scale structure: the general structure is of the third scale, consisting of an octahedron made of octahedrons lattices (second scale), which are composed of even smaller octahedrons (“cells”).

Cellular lattice structures have been at the forefront of lightweighting through AM due to their ability to tailor mechanical responses through the tuning of the topology, surface thickness, cell size, and cell density. It can be designed for specific performance characteristics and has numerous advantages due to their large surface area, low weight, regularly repeated structure, and open interconnected pore spaces [119]. The current AM techniques allow for their precise reproduction at both laboratory and industrial levels.

## 9. Aerospace and Car Racing Industries as Drivers of Lightweighting in Mainstream Automotive Vehicles

The lightweighting trend in the automotive industry is highly influenced by the entire transportation sector. In particular, aerospace and car racing industries are drivers of the technological development in mainstream automotive vehicles, reaching well beyond the subject of materials, for example, as seen in the radar-based driver assistance features of four-wheel steering.

Although the there are many differences between the aerospace and automotive industries, and both have their distinct paths of development, historically, aerospace material innovations continuously penetrated into the automotive industry.

As an expedition into technology transfer, serve the luxury and high-end performance automotive brands because the product cost and its affordability were not the primary constraints. With fewer regulations and cost considerations than in the mainstream automotive industry, car racing became an open test ground for changing industry boundaries and generating innovations that have far-reaching impacts on the future of mobility. When considering the fuel economy, in auto racing, this factor controls the difference between winning and not finishing the race. One of the reasons racecars are able to reach such impressive track times is because they are light. Thus, racecar designers routinely utilize lightweighting strategies to help make their cars fast.

**Figure 20:**
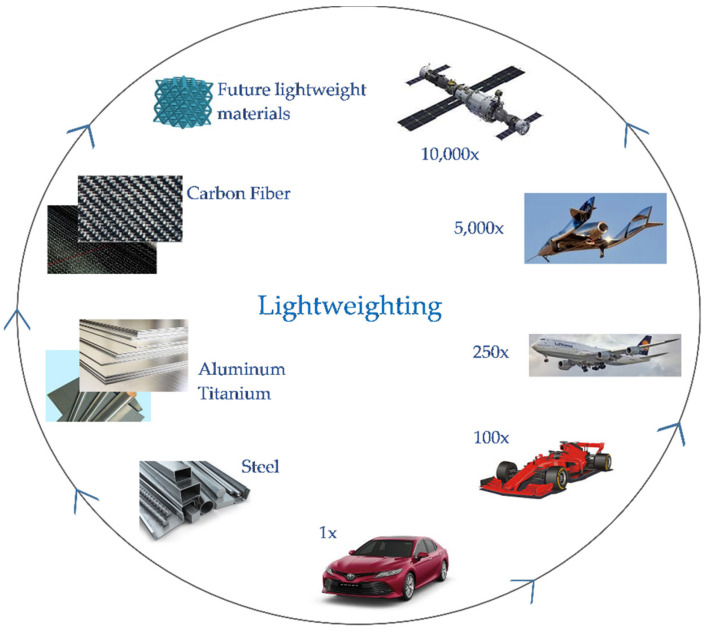
Schematics of the relative lightweighting cost in different sectors of transportation and the associated progress in lightweight materials.

The interconnection between aerospace and automotive industry is schematically portrayed in Figure 20, representing an updated version of the Ashby’s handbook explanation [120]. The value of lightweighting over the vehicle lifetime, expressed through arbitrary numbers, helps to understand at least the scale of magnitude. It should be kept in mind that while these values are useful as general guidelines, specific cases can sometimes land in unexpected regions of this chart and high-end racecars can utilize spacecraft-tier exchange constants.

As a challenge to overall technology, progress remains the material development line, as showed in this figure. During the past two decades, carbon-fiber composites have transformed aerospace manufacturing. Newer aircrafts, such as the Boeing 787 Dreamliner and the Airbus A350 XWB, are built mainly out of carbon fiber or other composite materials instead of heavier metals. Before carbon fibers, aluminum was used to build aircrafts, as it was lighter compared to steel. Although the carbon-fiber composite cost exceeds the cost of steel by up to two orders of magnitude, it is found in a variety of applications in luxury and high-end performance automotive brands.

At the top of the material progress chain of the Ashby’s illustration, the additive manufacturing is shown [120]. It is symbolic since it definitely eliminates many manufacturability constrains. However, from the perspective of time, it does not appear to be the ultimate solution to lightweighting. An increasing demand for lightweight materials led to an expansion of research regarding non-conventional alloying concepts in high-entropy compositions [121]. It is anticipated that a search for lightweight materials will soon become a part of the emerging concepts of development of novel materials for a low-carbon future; for instance, the accelerated materials discovery platform (MAP) aims to combine artificial intelligence, robotic systems, and high-performance computing to achieve autonomous experimentation [122]. For this reason, this spot in Figure 20 was left open to the novel ultimate lightweight materials likely to be developed in the near future.

## 10. Conclusions

The automotive lightweighting strategy is becoming the mature growth trend, driven by sustainability, cost, and performance, and creating an enormous demand for modern lightweight materials and design concepts. The lightweighting strategy is growing as a part of the circular economy and is the solution for both modern mobility and transportation; its objectives are not exclusively focused on the reduction of weight but also cover other aspects such as structural efficiency as well as economic and environmental impacts. It appears that the emergence of electric vehicles creates even more pressure on lightweighting.

In current lightweighting strategies, in addition to design, the materials represent the key part of the trend. A quest for lightweight materials creates many challenges and opportunities not only for existing conventional metallic alloys but also for novel strategies of achieving lightweighting goals through structural engineering, including metal-matrix composites, laminates, sandwich structures, and bionic-inspired archetypes.

Lightweighting design combined with the use of advanced lightweight materials leads to structural optimization, maximum weight reduction, and fulfilled required performance and safety standards. Manufacturability is still an important limitation to the design of lightweight structures but with progress in additive manufacturing, this constraint will gradually be eliminated.
